# Elder abuse and neglect: an overlooked patient safety issue. A focus group study of nursing home leaders’ perceptions of elder abuse and neglect

**DOI:** 10.1186/s12913-020-5047-4

**Published:** 2020-03-12

**Authors:** Janne Myhre, Susan Saga, Wenche Malmedal, Joan Ostaszkiewicz, Sigrid Nakrem

**Affiliations:** 1 Department of Public Health and Nursing, Faculty of Medicine and Health Sciences, Norwegian University of Science and Technology NTNU, Trondheim, Norway; 2 Centre for Quality and Patient Safety Research- Barwon Health Partnership, Institute for Healthcare Transformation, Deakin University, Geelong, Australia

**Keywords:** Elder abuse, Neglect, Patient safety, Long-term care, Nursing homes, Care managers, Leadership, Qualitative, Focus group

## Abstract

**Background:**

The definition and understanding of elder abuse and neglect in nursing homes can vary in different jurisdictions as well as among health care staff, researchers, family members and residents themselves. Different understandings of what constitutes abuse and its severity make it difficult to compare findings in the literature on elder abuse in nursing homes and complicate identification, reporting, and managing the problem. Knowledge about nursing home leaders’ perceptions of elder abuse and neglect is of particular interest since their understanding of the phenomenon will affect what they signal to staff as important to report and how they investigate adverse events to ensure residents’ safety. The aim of the study was to explore nursing home leaders’ perceptions of elder abuse and neglect.

**Methods:**

A qualitative exploratory study with six focus group interviews with 28 nursing home leaders in the role of care managers was conducted. Nursing home leaders’ perceptions of different types of abuse within different situations were explored. The constant comparative method was used to analyse the data.

**Results:**

The results of this study indicate that elder abuse and neglect are an overlooked patient safety issue. Three analytical categories emerged from the analyses: 1) Abuse from co-residents: ‘A normal part of nursing home life’; resident-to-resident aggression appeared to be so commonplace that care leaders perceived it as normal and had no strategy for handling it; 2) Abuse from relatives: ‘A private affair’; relatives with abusive behaviour visiting nursing homes residents was described as difficult and something that should be kept between the resident and the relatives; 3) Abuse from direct-care staff: ‘An unthinkable event’; staff-to-resident abuse was considered to be difficult to talk about and viewed as not being in accordance with the leaders’ trust in their employees.

**Conclusions:**

Findings in the present study show that care managers lack awareness of elder abuse and neglect, and that elder abuse is an overlooked patient safety issue. The consequence is that nursing home residents are at risk of being harmed and distressed. Care managers lack knowledge and strategies to identify and adequately manage abuse and neglect in nursing homes.

## Background

Little is known about elder abuse in nursing homes, and compared to research on other forms of interpersonal abuse, research about elder abuse in nursing homes is still in its infancy [1, 2]. Although no national prevalence data are available in any country internationally, high rates of elder abuse and neglect have been reported in nursing homes, including Norway [1, 3]. According to the World Health Organisation (WHO), elder abuse has been identified in almost every country where these institutions exist [4]. In the Toronto Declaration, WHO defines elder abuse as ‘a single, or repeated act, or lack of appropriate action, occurring within any relationship where there is an expectation of trust which cause harm or distress to an older person’ [5] p:3. Prevention of harm is a core principle in health care services and a leadership responsibility [6–8]. Nursing home leaders are legally and morally responsible for ensuring that required quality and safety standards are met [6, 9, 10]. The National Patient Safety Foundation (United States) defines patient safety as ‘freedom from accidental or preventable injuries or harm produced by medical care’ [10], p,2. This includes preventing elder abuse and examining the factors that foster an unsafe environment for both residents and staff [6, 7, 11]. Furthermore, elder abuse can be categorized according to type of abuse. The definition from ‘Protecting Our Future: Report from the Working Group on Elder Abuse’ (Ireland) includes physical, psychological, financial and sexual abuse, and neglect (Table 2) [12]. Abuse in nursing homes may also be categorized according to type of relation [1]; staff-to-resident abuse [3, 13], family-to-resident abuse [14, 15] and resident-to-resident abuse, also called resident-to-resident aggression [16, 17].

A recent meta-analysis of the prevalence of elder abuse in long-term care settings estimated a pooled prevalence of 64.2% of abuse perpetrated by staff in the past year, where psychological abuse and neglect had the highest prevalence [1]. A survey of 16 nursing homes in the central part of Norway found that 91% of staff had observed a colleague engaging in some form of inadequate care,

and 87% of staff reported that they themselves had perpetrated some form of inadequate care in the past [3]. Comparably, in a study from Ireland, Drennan et al. found that 57.5% of staff had observed one or more abusive behaviours from a colleague in the previous year [13]. Neglect and psychological abuse were the most commonly observed or perpetrated acts [3, 13]. Living in a nursing home may also mean sharing room and space with co-residents, and in recent literature, resident-to-resident aggression has been identified as a common form of abuse in nursing homes [16–18]. Lachs and colleagues revealed that 407 of 2011 residents from ten facilities had experienced at least one resident-to-resident event over one month observation, showing a prevalence of 20.2%, and the most common form was verbal abuse [16]. The literature about elder abuse in domestic settings shows that close family and friends can be perpetrators of abuse [15], but few studies have investigated the role of family members as perpetrators of abuse in nursing homes. A study from the Czech Republic found that nursing home staff had observed relatives participating in financial exploitation combined with psychological pressure on residents in nursing homes [14]. However, comparing findings in the literature on elder abuse in nursing homes is challenging because definitions and understandings of abuse can vary in different cultures, jurisdictions, and among health care staff, researchers, family members, and residents themselves [1, 2, 11, 19–21]. Different understandings of what constitutes abuse and its severity complicate detecting, reporting and managing the problem.

Nursing homes are complex social systems that consist of different participants, including staff, leaders, residents and relatives in constantly shifting interactions [22, 23]. The aetiology of abuse in nursing home settings is described as complex, comprising varying associations between personal, social and organisational factors [2, 24]. Nursing home residents often have complex care needs, dementia or other forms of cognitive impairment [25], display challenging behaviour [26], and depend on assistance in daily activities and care, all factors associated with a high risk of abuse and neglect [3, 13, 24, 27]. In Norway, 80% of nursing home residents have dementia, and 75% have significant neuropsychiatric symptoms such as agitation, aggression, anxiety, depression, apathy and psychosis [25]. Residents who display aggressive behaviour toward staff are at greater risk of experiencing abuse [13, 27, 28]. Findings in Drennan et al.’s Irish study revealed that 85% of the nursing home staff had experienced a physical assault from a resident in the previous year [13]. Aggressive behaviour has also been found to trigger resident-to-resident aggression in nursing homes [16, 17]. Related to organisational factors, there is an association between inappropriate environmental conditions for residents, low levels of staffing, and abuse and neglect [13, 14, 29]. As a result of this complexity, elder abuse in nursing homes is difficult to define precisely [11]. Within the literature, elder abuse in nursing homes is conceptualised as a specific form of institutional abuse [30] and a setting in which abuse and neglect take place [14], since rules and regulations in institutions can be abusive themselves, e.g., deciding residents’ sleeping and meal times, the use of restraint, and shared living spaces with other residents.

Good leadership plays a key role in developing staff’s understanding of residents’ needs [31, 32] and creating a strong safety culture of respect, dignity, and quality [6, 7, 9, 33]. The importance of leadership in developing a patient safety culture is highlighted in a report from the National Patient Safety Foundation [10]. In Norway, governmental strategies to improve leadership and safety culture have been launched, such as the Patient Safety Programme and a system for monitoring health services using quality indicators [34]. Leadership is defined as a process whereby a person influences a group of individuals to reach a common goal [35], such as a strong safety culture. The safety culture of an organisation is defined as ‘the product of individual and group values, attitudes, perceptions, competencies, and patterns of behaviour that determine the commitment to, and the style and proficiency of, an organisation’s health and safety management’ [10, 36] p:23. This includes detecting situations that can be harmful to residents. However, several studies have shown that underreporting of abuse and neglect is a significant problem [1, 37, 38]. Residents’ own inability to communicate about the abuse or their fear of repercussions and retaliation are important factors of underreporting [1, 2]. Therefore, staff should be able to recognise and report situations that can be perceived as harmful or distressful from the perspective of residents. However, a systematic review of staff’s conceptualisation of elder abuse in residential care found that staff were often uncertain about how to identify abuse, especially psychological abuse and caregiver abuse and neglect [39]. Despite the vast knowledge that exists about the importance of leadership, nursing home research has not yet paid much attention to the role leaders play regarding identifying elder abuse. Consequently, there is a gap in knowledge about elder abuse from the perspective of nursing home leaders. Knowledge about nursing home leaders’ perceptions of elder abuse and neglect are essential because their understanding of the phenomenon will affect what they signal to staff as important to report and what they investigate to create a safe and healthy environment. To our knowledge, this is the first study that seeks to understand the nature of elder abuse from the perspective of nursing home leaders.

## Methods

### Aim of the study

The aim of the study was to explore nursing home leaders’ perceptions of elder abuse and neglect.

### Design

The present study is part of a larger study funded by the Research Council of Norway (NFR), project number 262697. A qualitative exploratory design with focus group interviews was conducted to gain greater insight into this important but poorly understood topic. Qualitative methods provide knowledge about people’s experience of their situation and how they interpret, understand and link meaning to events [40, 41]. In focus group interviews, group dynamics allow the questions to be discussed from several points of view, and the group’s dynamics can create new perspectives and opinions during the discussion [42]. This study follows The Consolidated Criteria For Reporting Qualitative Research (COREQ) (Additional file 1).

### Settings

In Norway, approximately 39,600 residents live in nursing homes (12.9% of the population > 80 years), and their mean age is 85 years [43]. These nursing homes are mainly run by the municipalities and financed by taxes and service user fees. Residents pay an annual fee equal to 75% of the resident’s national age pension. In addition, residents may pay an additional fee if they have income of their assets, but with an upper limit decided by the government. However, the payment cannot exceed the actual expenses of the institutional stay [44].. Management of care in Norwegian nursing homes is regulated by ‘the regulation of management and quality improvement in health care services’ [45]. The regulation focusses on the leader’s responsibility to ensure that residents’ basic needs are satisfied. This includes the leader’s responsibility to ensure there is a system in place to monitor residents’ overall quality and safety and to create a safety culture that detects situations and factors that can cause harm to residents and staff [45].

Each nursing home is required to have an administrative manager, called the nursing home director, and some nursing home directors lead more than one facility. In addition, each nursing home has ward leaders and quality leaders, and in some municipalities, a service leader. Together, individuals in these leader roles form the leadership team in each nursing home [46]. The ward leader is a registered nurse (RN) who supervises and manages staff. Ward leaders are also responsible for budgets in their own wards and the quality of care for residents. There are often several wards and ward leaders in each nursing home. The quality leader is an RN who monitors the overall quality of care in the nursing home in collaboration with the ward leaders. The service leader supervises and manage service staff members who are in contact with nursing home residents (e.g., activity coordinators, cleaning staff and kitchen staff) and is also responsible for the budget related to his or her staff. Individuals employed in one of these leader positions provide the closest level of leadership to staff and residents but are not part of the daily direct hands-on care of residents. There is no national requirement regarding formal leader education to be employed in these leader positions, but leader education is a high priority in many municipalities. These individuals often have lengthy experience as RNs or have previous leader experience.

### Sample

The study sample was recruited from 12 nursing homes in six municipalities in Norway. Inclusion criteria were a person who: (a) was employed in a leader position as ward leader, quality leader, or service leader in a nursing home, and (b) was employed full time in the leader position. The inclusion criteria were chosen because these individuals directly affect quality and safety in the nursing home, as they are the closest level of leadership to the staff and residents. Purposive sampling was initially used to ensure that participants recruited could see the phenomenon from the perspective of a leader. During the data collection, each municipality and its nursing home leaders were recruited using a step-wise approach, as we were seeking to get a theoretical sampling until saturation of data was achieved [40, 41]. A total of 28 individuals participated in the study, 23 participants were ward leaders, two participants were quality leaders, and three participants were service leaders. However, in this study, all 28 participants are named ‘care managers’. Characteristics of the participants are presented in Table 1.

**Table 1:** Demographics of the sample (*n* = 28)

Background characteristics	Number (%)
**Age (years)**	
30–39	6 (22)
40–49	11 (39)
≥ 50	11 (39)
**Gender**	
Female	25 (89)
Male	3 (11)
**Number of beds managing:**	
0	5 (17)
10–19	8 (29)
20–29	8 (29)
≥ 30	7 (25)
**Number of staffs managing:**	
0	2 (7)
10–29	9 (33)
30–49	11 (39)
≥ 50	6 (21)
**Years in this position**	
0–4	20 (71)
5–9	7 (25)
≥ 10	1 (4)
**Total working experience as a leader in years**	
0–4	11 (39)
5–9	6 (22)
≥ 10	11 (39)
**Formal leader education**	
0	1 (4)
0,5–1 years course	18 (64)
1–2 years course	3 (11)
Master’s Degree	6 (21)

### Recruitment and data collection

Participants were recruited over a period of six months, from August 2018 through the end of January 2019. A recruitment email was sent to health care managers in 11 municipalities in both urban and rural areas. Health care managers from five municipalities stated that they could not find time to participate in the study, while six health care managers accepted the invitation. Thereafter, a second recruitment email was sent to all nursing home directors in these six municipalities. The email included an invitation letter, which the nursing home director forwarded to all individuals employed in a leader position at their nursing homes. Six focus group interviews were conducted, with three to six participants in each group. The focus groups were composed as follows: one focus group with three participants; two focus groups with four participants; one focus group with five participants; two focus groups with six participants.

**Fig. 1:**
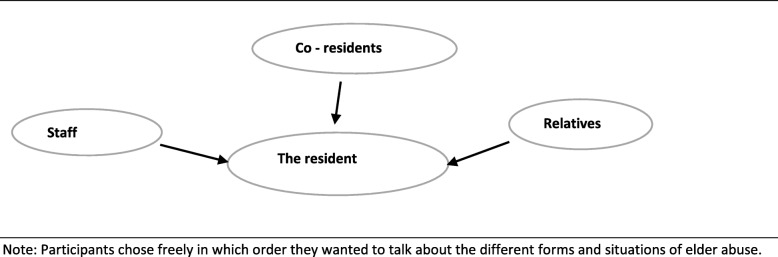
Model of interactions where abuse can occur as used in the interviews

All six focus group interviews took place in a meeting room in a nursing home in the participating municipalities. Each focus group interview lasted approximately 90 min. All participants gave informed written consent before the interviews started. Two researchers carried out the interviews. JM was the moderator in all six interviews, SN was co-moderator for two group interviews, and SS was co-moderator in one group interview. In the other three interviews, two researchers from the larger research team were co-moderators. During the introductory information about the focus group interview, we presented a figure (Fig. 1), and asked participants about their experience and thoughts on the topic of elder abuse from health care staff, co-residents or relatives. Participants were encouraged to speak freely. However, during the first interview, we experienced that participants were not familiar with the topic. To explore the topic in the ensuing interviews, the moderator gave the participants keywords from the categorization of abuse (e.g., abuse can be described as physical, psychological, sexual, financial, or neglect) (Table 2) [12]. We found that this helped the participants reflect, and they subsequently came up with examples of abusive situations they had heard about or witnessed. During the process of data collection, we further compared our experiences in interview one with interview two, which is in line with the constant comparative method [40]. This led to including keywords in the interview guide to ensure that all topics were covered (Additional fil 2). To ensure the credibility of an open thematic understanding of participants’ experiences and diminish bias by presenting the keywords, we were conscious about letting the participants speak freely about their experiences and thoughts on this topic. Moreover, they were not given any definition of abuse or examples related to these keywords (Table 2) [12]. The participants freely decided in which order they wanted to talk about different forms and situations of elder abuse. All interviews were recorded and transcribed verbatim, retaining pauses and emotional expressions.

**Table 2:** Operational definitions of abuse and neglect in residential settings [12]

Five areas of abuse and neglect	Abusive actions
*Physical Abuse*	Hitting, slapping, pushing, kicking, misuse of medication or restraint.
*Psychological abuse*	Emotional abuse, threats of harm or abandonment, deprivation of contact, humiliation, blaming, controlling, intimidation, coercion, harassment, verbal abuse, isolation or withdrawal from services or supportive networks.
*Sexual Abuse*	Rape and sexual assault or sexual acts to which the older adult has not consented, or could not consent, or into which he or she was compelled to consent into which he or she was compelled to consent.
*Financial Abuse*	Theft or the misuse or misappropriation of property or possessions.
*Neglect*	Ignoring medical or physical care needs, failure to provide access to appropriate health care, social care or educational services, withholding of necessities of life, such as medication, adequate nutrition and heating.

### Data analysis

A constant comparative method with a grounded theory approach was used. This allowed us to generate a thematic understanding of elder abuse through an open exploration of the experience described by nursing home leaders [40, 41]. The constant comparative method facilitated possible identification of themes and differences between individuals and cases within the data [40]. Our analysis started right after each interview, where the first author listened to the recorded interview. Memo writing was then used through the whole process of data collection and analysis and served as a record of emerging ideas, questions and categories [41]. Next, in line with the constant comparative method, open line-by-line coding of the transcribed interviews was performed [40, 41], since we wanted to capture the meaning from the participants’ perspectives as they emerged from the interviews. The codes were compared for frequencies and commonalities and then clustered to organise data and develop sub-categories. The sub-categories were examined to construct the final categories and main theme. To add credibility and diminish researcher bias, two researchers (JM and SN) coded the transcribed interviews independently. During the analysis process, the authors held several meetings where codes and their connections were discussed until consensus was reached. To ensure that the emerging categories and themes fit the situations explored, the researchers went back and forth between contextualization, data analysis and memo writing [40]. An example of the analysis process is shown in Table 3.

**Table 3:** Example of data analysis in the category “abuse from co-residents”

Sub- Categories	Code	Meaning unit
Common	Resident-to resident aggression are common	*We have very often residents that are both physically and psychological aggressive towards other residents.*
Resident - to resident aggression as normalized	Difficult to do something with resident- to resident aggression	*I think it is due to the cognitive failure, so then it is not an abuse, because it doesn’t help to just talk to the resident.*
	Resident-to-resident aggression a big part of everyday life in nursing homes	*We may have a little thick skin in relation to where the limit goes for what we accept. Because it is such a big part of our everyday life that it became normal in a way.*
	Normal behaviour from people with dementia	*When we have focus on dementia, it becomes normal for us to see such behaviour.*
Hitting	Physical abuse – hitting when trespassing a resident rom	*We had a patient who was hit and beaten by the same resident several times. The resident walks into his room and simply knocked him down, and that is a despair.*
Verbal abuse	Psychological abuse – verbal abuse normal behaviour for people with dementia	*Then we have residents with frontotemporal dementia who just acts in that way, they just verbally offending others, but it is their way of behaving.*
Violation of resident’s privacy	Psychological abuse – violation of resident’s privacy when trespassing into another resident’s room	*Trespassing into another residents’ room that happens a lot, but it’s a violation of their privacy, and if the resident can’t speak or is cognitive impaired, they may be unable to tell if something is happening.*
Stealing things	Financial abuse – stealing things	*They steal things from each other’s room, yeas that happened.*
Sexual assault	Sexual abuse – sexual assault and an ethical dilemma	*We see sexual approaches or that they forgot that they are married and find each other instead. But that is more a dilemma than an assault …. or maybe it can be an assault… well I don’t know.*

### Ethical consideration

Ethical approval for this study was given by the Norwegian Centre for Research Data (NSD), Registration No: 60322. Each participant signed a written consent form after receiving oral and written information about the study. All identifiable characteristics are excluded from the presentation of data to ensure the anonymity of all individuals.

## Results

The main theme, ‘Elder abuse in nursing homes, an overlooked patient safety issue’, found in this study indicates an overall lack of awareness of elder abuse and its harm among care managers. Three analytical categories emerged from the analyses: 1) Abuse from *co-residents – ‘A normal part of nursing-home life’*, 2) Abuse from *relatives – ‘A private affair’*, and 3) Abuse from *direct-care staff* – *‘An unthinkable event’.* Since there were no remarkable differences in care managers’ experiences, we present results without differentiating the participants. Below, we describe each category, together with examples of forms of abuse and neglect. These examples are used to describe the care managers’ perceptions of elder abuse and neglect (Table 4).

**Table 4:** Examples of forms of abuse as described by care managers

	*Co- residents* ***“A normal part of nursing home life”***	*Relatives* ***“A private affair”***	Direct Care staff ***“An unthinkable event”***
* Physical abuse*			
Hitting, kicking, pushing, and throwing things	X		X
Rough handling		X	X
Use of force or restrain		X	X
*Psychological abuse*			
Verbal abuse	X	X	X
Violation of resident’s privacy	X		X
*Financial abuse*			
Stealing or destroying a resident’s assets	X	X	X
*Sexual abuse*			
Sexual assault	X		X
*Neglect*			
Neglect of user participation		X	X
Health care neglect			X

### Abuse from co-residents – ‘A normal part of nursing-home life’

Resident-to-resident aggression was described as the biggest issue related to abuse in nursing homes and a daily challenge for the participants: ‘*That is what I also see, that co-residents are the biggest challenge regarding this topic’* (Group 2). The main cause of resident-to-resident aggression reported by care managers was symptoms of dementia, especially in the initiator, but also in the victim. The care managers expressed that they did not know how to address this problem. As one said, ‘*It happens because of the cognitive failure, so yes. But, at the same time, it is also difficult to do something about it’* (Group 2). Some care managers also stated that the risk of harm caused by resident-to-resident aggression was something residents must accept when living in a nursing home: ‘*There is a predictable risk, when living in nursing homes, [of] such incidents; there is a foreseeable risk that this will happen’* (Group 5)*.* This demonstrates that resident-to-resident abuse is normalized.

Care managers considered physical abuse to be the most serious form of resident-to-resident aggression, often leading to visible harm and despair. At the same time, all care managers had examples of residents who had been beaten, knocked down, or kicked by co-residents.

*‘We have one resident now that is beaten a lot by the other residents. It’s a little extreme, but I think that such things can happen quite often in dementia care because, as in this case, the resident being beaten is not silent for a minute. She speaks and yells all day, and the other residents become annoyed since she disturbs them’* (Group 4).

Care managers described psychological abuse as acts of ‘everyday bullying’ and threats made among residents. They interpreted these situations as a normal consequence of the dementia disease in the individual resident. One care manager noted, ‘*What I think is the challenge is the everyday bullying. It is seen as normal behaviour for that group of residents’* (Group 1). When discussing psychological abuse connected to co-residents, all care managers provided examples of residents trespassing in other residents’ rooms. They interpreted this behaviour as a violation of residents’ privacy. At the same time, it was perceived as normal since it happened quite often. The care managers also reported that when residents trespassed and entered another resident’s room, the risk of other forms of abuse such as financial abuse increased. One care manager remarked*, ‘We have some challenges related to residents who enter other residents’ rooms and destroy or take other residents’ possessions. It can be pictures and different things’* (Group 3).

Related to sexual abuse by co-residents, all care managers had examples of residents who had shown sexual interest in another resident. The care managers viewed this sexual interest as an ethical dilemma for them. On the one hand, they want residents to have a healthy sex life in the nursing home, but on the other hand, this is difficult when a resident has dementia and may not be competent to give consent. Several care managers experienced that what seemed to be voluntary sexual interest between residents could not be that, after all:

*‘In that situation, she was very interested in him, and he was very interested in her. And it was like, yes, they were in the room together and so on. I remember it as very, very difficult because she often had a lot of pain. I do not know if there was penetration, but it was, in any case, an attempt, yes, it may as well have been that too. I had a lot of trouble because I was unsure whether she understood what happened and who it was happening with because it was often very difficult for her after they had been in the room together. I remember it as a huge ethical dilemma. But I never thought that it was a sexual*. *.*. *that it was an assault or something. But, right now, I think it was’* (Group 5).

During the focus group discussion, care managers reflected on the complexity of letting residents express themselves sexually and the risk of sexual assault. From their statements, it was clear that they had not reflected on this topic earlier. A summary of forms of harmful situations related to resident-to-resident aggression reported by participants is presented in Table 4.

### Abuse from relatives – ‘A private affair’

Abuse directed towards residents from their relatives was reported to be a particularly difficult problem. According to the care managers, relative-to-resident abuse was often hidden, occurring behind private closed doors when a relative was visiting the resident. Therefore, participants described it as difficult to discover and associated mainly with the private relationship between the resident and his or her relatives:

‘*It is very difficult. It is a relative who is going to visit her mother in the nursing home, she closes the door to the room and wants to be there alone with her mom, and we have very large rooms, so we thought they were having a nice time inside the rom. But then we discovered that the mom had some bruises, and then we understood that things were happening’* (Group 3).

Not all care managers had knowledge of or experience with relative-to-resident abuse, which highlights the private nature of these forms of abuse. Abuse from relatives was viewed as being linked to past family conflict, which continued inside the nursing home. The care managers deliberated over the extent to which they should interfere in the private relationship when they suspected this form of abuse. They reported that the problem was knowing what to do and when and how to interfere, especially when the resident has dementia or another form of cognitive impairment. One care manager remarked, ‘*It is very difficult. I have a patient who may not be competent to give consent. So, I have a responsibility I must take, but I think it’s challenging to know what to do’* (Group 2). Cases where the resident clearly did not want anyone in the nursing home to know about the abuse or to do anything about it and just wanted to maintain the relationship with his or her family member despite the abuse were reported to be particularly difficult. The care managers expressed that they lacked a strategy or authority in these situations, and harm to the resident being exposed was accepted.

‘*But it is not always that the resident wants us to do something, either. It may have been this way for a long time, and then, maybe it’s okay then. Well, I don’t know’* (Group 5).

Physical and sexual abuse from relatives was regarded as the most hidden form of abuse from relatives. Some care managers provided examples of physical abuse, but none had experienced sexual abuse. However, all care managers commented that when it happened, it took place behind private closed doors. In addition to past family conflict, abuse from relatives was often related to mental problems and/or drug abuse issues. One care manager said, ‘*I have experienced some older people who have children with drug issues and such things. And it is in those cases, I have experienced physical abuse towards residents from relatives’* (Group 4). Related to physical abuse from relatives, care managers also reported situations where a relative forced the resident to, for example, eat, get dressed, wash and groom, or exercise. These situations were linked to unrealistic expectations in relatives, and not trusting the staff is doing a good job.

*‘After her husband had been there, we saw that she was so red around the cheek. We then found out that the husband squeezed her mouth open and poured cream into her’* (Group 3).

Care managers viewed psychological abuse from relatives as disrespectful communication with the resident. A participant stated, *‘We experience that relatives can be quite disrespectful to their loved ones. But, at the same time, it may have been this way their whole life’* (Group 6).

Care managers expressed that financial abuse from relatives was a common occurrence. They cited examples of stealing money from residents, threatening residents in order to get money from them, and unauthorized use of a resident’s finances. One participant stated, ‘*What I see most from the relative’s part is financial abuse. It is very common, actually’* (Group 1). Relatives’ economic problems were reported to be a causal factor related to financial abuse. At the same time, care managers indicated that financial problems and financial exploitation by relatives were private issues, and as such, they were reluctant to interfere.

Related to neglect, care managers described that some relatives made decisions on behalf of the resident without considering what the resident wanted and needed or would agree upon. Care managers stated that sometimes the health care staff also disagreed with the relative’s decision. One care manager noted, ‘*We have situations where relatives make decisions on behalf of the resident, which we do not agree upon, and which we might think the resident would not agree upon either’* (Group 3*).* Care managers also described experiences of relatives who refused to allow a resident to buy items the care managers considered necessary and not provided by a nursing home. These could be things such as clothes, hairdressing services, or podiatry, but it could also be related to taking part in activities that cost money. A care manager remarked:

‘*I have a resident who called her son to ask if she could go to a podiatrist because she really needed it, but her son refused and said she has no money for that’* (Group 5).

Thus, because of neglect by their relatives, residents might go without necessities of daily living and may not be able to participate in activities they would like to take part in. A summary of forms of harmful situations related to relative-to-resident abuse reported by participants is presented in Table 4.

### Abuse from direct-care staff – ‘An unthinkable event’

When care managers were prompted to talk about staff-to-resident abuse, they reframed the discussion to focus on the verbal and physical aggression they commonly experienced from nursing home residents. They interpreted aggression directed toward them as a risk to their health and safety. Moreover, they stated this phenomenon was a daily concern. One noted, ‘*We have the opposite focus in our units. We focus on staff being subjected to abuse by residents’* (Group 2). Several care managers also indicated that they understood that staff could become stressed and frustrated in their relationship with an aggressive resident:

*‘We have a case that is extremely difficult, where there are many violations against staff by a resident. And then, to be in such a situation where you can quickly retaliate*. *.*. *this is difficult’* (Group 6).

Despite this, care managers expressed that elder abuse was not a topic they talked about in their daily work at the nursing home. They indicated that they wanted to trust the employees. Therefore, abuse from staff was difficult to talk about and almost unthinkable to them. One care manager said, *‘I think that no one who works in the nursing home started there just to be able to hurt someone, and that is perhaps why this is such a sensitive and difficult topic’* (Group 5)*.* The word ‘abuse’ was also reported to be a very strong term and mainly related to intentional physical acts. However, in the discussion, care managers also included unintentional acts in their examples of elder abuse and expressed that, to some degree, it could be difficult to know the full intention of a staff member’s actions. At the same time, they emphasised that staff’s intentions were mainly good, and therefore abuse was unthinkable:

*‘Everyone who works in a nursing home is motivated by and has a desire to help someone. So, most of the [incidents] of abuse by staff*.*.*.*I think it may be those with a good intention at the heart of it. [For instance, thinking] “I thought he should have a shower, but I forgot to ask”* (Group 5).

Care managers discussed examples of the use of physical and chemical forms of restraint and rough handling during care. Utilization of restraints and dilemmas related to their use was discussed in all focus groups, and care managers pointed out that the staff are sometimes compelled to use both physical and chemical restraints to help or protect the resident:

*‘I think in relation to, well it is really both physical and psychological abuse. I think of cases, especially at night, where there is low staffing and many residents with aggressive behaviour, where it may be chosen to lock some residents into their rooms to prevent them from being exposed to abuse from co-residents so the staff can deal with the situation, but it is abuse to be locked inside’* (Group 2).

Rough handling was something that all care managers had experienced. This was thought to be mainly unintentional and something that could happen when caring for residents with aggression or those who resist care. Care managers expressed that, to define it as abuse, it had to be significant, or there needed to be visible signs of such handling, such as bruising. At the same time, the care managers also pointed out that residents in nursing homes often bruise easily, and it can be difficult to determine whether such marks are related to abuse:

*‘Sometimes, we saw that she was so easy to bruise, and sometimes we clearly noticed hand marks on the bruises around her body. But it can be enough that you handle someone a little hard, and in the old ones, then they get bruises, although it can also indicate that there has been resistance, right. But then this happens all the time’* (Group 4).

Psychological abuse from staff members was linked to verbal abuse. Care managers cited examples of yelling at a resident in anger, speaking to a resident in a disrespectful tone, or being rude, which allegedly occurred in relation to resident-to-staff aggression. When discussing psychological abuse, some care managers also provided examples of violations of residents’ privacy by staff members, such as discussing residents’ health care issues and challenges in public areas in the nursing home:

*‘If there has been a resident with a rejection of care responses, for example, that has been difficult to cooperate with, then that frustration can be expressed in public areas with other residents present. Without caution by staff, this is something other residents are going to hear’* (Group 5).

Financial abuse was thought to be related to stealing money or destroying a resident’s property. At the same time, care managers reported that their nursing home policies do not allow residents to keep much money in their rooms in order to protect residents from financial abuse by staff, visitors, or others, and hence, financial abuse from staff rarely happened. One said, *‘Financial abuse only happens if the residents have money laying around’* (Group 1).

When talking about sexual abuse, care managers offered examples of residents who stated that they were sexually assaulted by staff members. These were often female residents who expressed that male staff had sexual intentions towards them during care. At the same time, care managers reported that such statements from residents could be part of the dementia disease, and that resident could have hallucinated the abuse. Care managers indicated that sexual abuse by staff was unthinkable to them:

*‘Sometimes, older people with cognitive impairment say things that we can become uncertain about. They say things, but we can’t be sure there has been an assault. Often, we think that it has not happened. It’s about us knowing them; they say a lot of these things and are very sexually oriented’* (Group 4).

Even so, a few care managers mentioned examples of sexual abuse by staff a long time ago that had been reported to the police, and the staff member was convicted.

Related to neglect, care managers reported that staff often did things for residents to save time instead of letting them do it independently. They also reported being aware that, in many situations, staff members do not pay attention to residents’ wishes and thereby neglect to include them in decisions concerning daily life in the nursing home. One care manager noted, ‘*It says on the duty list that you should shower today, so you should shower, even if you might say, “No, I don’t want to.” So, yes, it is your turn today’* (Group 3). Another form of neglect by staff was reported to be linked to health care neglect. Care managers referred to events such as not helping a resident with needed health care, giving a resident an incontinence product instead of helping them use the toilet, not calling for medical help when needed, and not following up on medical conditions:

*‘To put on a pad instead of following the patient to the toilet, for those who still manage to use the toilet themselves*.*.*.*that can happen’* (Group 6).

The care managers reported that, because of low financial resources, staff must prioritize their work and tasks every day. For this reason, situations not specifically related to medical treatment and physical or health outcomes were given lower priority. This reprioritization was framed as acceptable and was not defined as neglect. One said, ‘*It is about our time. So, no, we don’t have time for you or that need is not important. It is about what we have to prioritize’* (Group 6). A summary of forms of harmful situations related to staff-to-resident abuse reported by participants is presented in Table 4.

## Discussion

The aim of the study was to explore nursing home leaders’ perceptions of elder abuse and neglect. We found that most of the care managers were not explicitly aware of elder abuse in their daily work. However, when given keywords, they all came up with examples of situations they interpret as harmful or distressful to residents. This shows that care managers need time to reflect on complex aspects of care to become aware of abuse and neglect as a safety issue. At the same time, our findings revealed an ambiguity in the care managers’ examples. The situations, on the one hand, were described as harmful. On the other hand, they were rationalized as care managers attempted to excuse why it was happening. Three main categories are described in the finding: *Abuse from co-residents – ‘A normal part of nursing-home life’, Abuse from relatives – ‘A private affair’, Abuse from direct care staff – ‘An unthinkable event’.* These findings indicate that this cohort of nursing home care managers lack awareness of the abuse they observe or hear about. Particularly, these findings demonstrate that harm or distress to residents caused by abuse are an overlooked patient safety issue in these nursing homes.

Findings revealed that resident-to-resident aggression is a common form of abuse in nursing homes and a daily challenge. There is a high prevalence of residents with neuropsychiatric symptoms of dementia, including aggression, agitation and psychosis in nursing homes [25, 26]. These symptoms impact on co-residents and staff safety, and resident-to-resident aggression is the most common form of abuse in nursing homes [16, 17]. However, our findings revealed that harm resulting from resident-to-resident aggression was perceived as normal. This raises the question of whether care managers perceptions place the responsibility on the resident, without accounting for the complexity in the aggressive behaviour and the responsibility of the organization [22]. It is worth noting that in resident-to-resident aggression, both residents can suffer harm, since the initiator is likely to be confused and usually not responsible for the acts. For the victim, resident-to-resident aggression has both physical and psychological consequences [47]. However, previous research has also indicated that abusive behaviour can be understood as less abusive when the victim has dementia, and for that reason it is often not reported [17, 48]. Recognising that aggressive behaviour has a multifactorial aetiology, best practice recommendations [49] and research evidence [50, 51] call for a comprehensive biopsychosocial approach that investigates the resident’s unmet needs, medical conditions, environmental factors, and interactions between residents and caregivers and a tailored response [49]. Care managers’ perceptions of resident-to-resident aggression as normal and a foreseeable risk, places residents at risk and is also a failure to deliver much needed care to the initiator.

With respect to relative-to-resident abuse, findings demonstrate that care managers perceive negative events resulting in harm or distress as a private affair between the resident and his or her relatives, and that is difficult to intervene. Similarly, to resident-to-resident abuse, this indicates that the care managers place the responsibility of the observed abuse on the relationship between the resident and his or her relatives, without accounting for the complexity and their own responsibility in these situations. Care managers examples of relatives who force a resident to eat due to unrealistic expectations and distrust in nursing home staff’s care reveals that care managers find it difficult to interact with families. This finding points to potential communication difficulties between staff and resident’s relatives that could adversely affect the resident [52, 53]. A Norwegian study that investigated quality of care from the perspective of families in long-term care found that family members saw themselves as an important link between staff and the resident, and an essential voice regarding the resident’s needs and wishes [53]. However, given the nature of the nursing home and the complexity of its organization and routines [22, 23], it can be difficult for someone outside the organization to judge what is and is not adequate clinical practice. Collaboration and communication with the residents and their relatives depend on how the culture in the nursing home view these interactions; the relatives with right to an opinion, or professional as experts and in control [6, 22, 52]. This will in turn affect the quality and safety of the care that is delivered to the residents.

Although some care managers had experience of staff-to-resident abuse within all abuse categories, it was also difficult for them to admit to this form of abuse, and it was viewed as an ‘unthinkable event.’ Instead, care managers were mostly interested in talking about resident-to-staff aggression which they emphasised was a larger problem in their nursing homes. Resident- to-staff aggression can cause physical and psychological harm to staff, reduced job satisfaction, stress and burnout, emotional reactions including sadness, guilt and helplessness [28]. However, resident-to-staff aggression may also lead to reactive abuse and neglect, due to frustration in staff member being exposed to aggression [11, 13, 27, 28]. Findings in the present study demonstrate that care managers lack awareness of the staff’s reactive responses to aggression from residents. This might raise the question if they perceive staff as victims in these situations and that abuse from staff is understandable. Unprovoked or intentional abuse towards a resident therefore is unthinkable with justification in their trust to the staff.

Difficulties in defining abuse in nursing home settings have been found in studies that include staff’s perceptions [39, 54], where abusive situations are seen as normal in the nursing home culture [17, 33, 39, 55]. However, these studies did not specifically focus on care managers’ or leaders’ understandings. Our study reveals important information related to detection and management of abuse in nursing homes, since care managers’ perception of abuse affects what they signal to staff as important to report. Care managers have the opportunity to influence the culture and care practice in the nursing home and are responsible for setting policies for the staff, it is therefore essential that they are aware of and able to face situations that constitute potential harm to the residents. But, to be able to define situations that can be experienced as harm and distress, it is essential to see situations from the perspective of the residents. Harm and distress are defined differently from the point of view of the one who causes the harm [39, 54], the one observing or hearing about it [14], or the one who experiences a situation of harm or distress [20, 21]. Our findings indicate that the care managers had difficulties in seeing potential harm caused by abuse and neglect from the perspective of the residents. Leaders’ abilities to promote a safety culture for both the resident and staff are linked to their leadership skills, knowledge of the resident’s needs and their capacity to implement effective safety care practices [6, 31, 32]. Care managers’ lack of awareness in identifying and following up on abuse will necessarily affect the safety culture in the organisation and, in the end, clinical outcomes such as quality and safe care for the residents [6, 10, 56].

A recent Norwegian study found that communication, openness and staffing were significant predictors of staff’s overall perception of patient safety in nursing homes, yet the nursing home staff scored low on these dimensions [56]. This finding aligns with our study, which revealed that care managers find it difficult to distinguish between prioritising and patient neglect. Low financial resources and low staffing can affect the perception of what constitutes harm and safety in the nursing home culture. Low finances, combined with the complexity of residents’ needs, the complex organisation, and demands for improved outcomes, puts great pressure on nursing home leaders [22, 57]. The ambiguity in their examples can be understood as an attempt to rationalize abuse and diminish their personal and professional accountability. People in complex social systems will try to make sense of tasks and orders by adapting to internal and external demands [22, 23]. Health care policies that mandate efficiency, cost saving, and nursing home care managers’ focus on prioritising contribute to lowering the limit for what is perceived as quality and safety, resulting in low quality and unsafe environment as the norm and accepted in nursing homes.

### Strengths and limitations of the study

A strength of this study is that it involves participants who are in leader positions in different nursing homes and municipalities in Norway, which could increase the transferability of these findings. The research team consists of members from two countries, all with broad research experience, which contributed to multiple perspectives and discussions during analyses of the data. This strengthens the trustworthiness of our findings, and the credibility of the research. Three of the authors have worked several years in nursing homes as care managers, but none of those nursing homes participated in this study. The researchers’ backgrounds as care managers has both advantages and disadvantages. A variety of aspects of participants’ experiences was discovered by posing in-depth questions that might not have been possible without the background knowledge. However, the background knowledge can influence the type of follow-up questions that were asked. To counterbalance this possible bias, two researchers were always present during the interview, and the analyses were also independently coded by two researchers (JM and SN). Each focus group consisted of three to six participants, which can be perceived as small groups and a limitation. However, the participants gave a rich description of the phenomenon. Therefore, we decided to include data from the smallest groups.

The examples of abuse and neglect our participants described in the present study could be second-hand information because leaders are not always part of the direct hands-on care residents receive. At the same time, this study has sought to understand the nature of elder abuse from care managers’ perspective, which is of great importance due to their responsibility for creating a safe environment for both residents and staff. Even though the examples are second-hand information, the findings are representative of the care managers’ perceptions of the information and what we thought was important to study.

## Conclusion

Many nursing home residents have dementia, neuropsychiatric symptoms, and complex needs, which increases the risk of their being exposed to abuse and neglect. At the same time, little is known about the nature of elder abuse in nursing homes and compared to research on other forms of interpersonal abuse, the study of elder abuse in nursing homes is still in its infancy. Care managers influence the culture and care practice in nursing homes and set policies for staff. Knowledge about their empirical understanding of the phenomenon is important to form more effective intervention and prevention strategies. The present study shows an ambiguity in the nursing home leaders’ examples of abuse and neglect. On the one hand, the situations were described as harmful. On the other hand, they were rationalized with an attempt to excuse their occurrence. Our study revealed that elder abuse and neglect is an overlooked patient safety issue in nursing homes. Care managers lack knowledge and strategies to identify and adequately manage abuse and neglect in nursing homes, and this warrants further research.

## Supplementary information

**Additional file 1.** COREQ checklist.

**Additional file 2.** Interview guide.

## Data Availability

The datasets generated and/or analyzed during the current study are not publicly available due to format of the data not allowing for completely anonymizing data but are available from the corresponding author on reasonable request.

## Abbreviations

NFR: Research Council of Norway
RN: Registered Nurse
WHO: World Health Organization

## References

[1] Yon Y, Ramiro-Gonzalez M, Mikton CR, Huber M, Sethi D. The prevalence of elder abuse in institutional settings: a systematic review and meta-analysis. Eur J Pub Health. 2018. 10.1093/eurpub/cky093 PMC6359898 29878101

[2] McDonald L, Beaulieu M, Harbison J, Hirst S, Lowenstein A, Podnieks E (2012). Institutional abuse of older adults: what we know, what we need to know. J Elder Abusw Negl.

[3] Malmedal W, Ingebrigtsen O, Saveman BI (2009). Inadequate care in Norwegian nursing homes--as reported by nursing staff. Scand J Caring Sci.

[4] Krug E, Dahlburg L, Mercy J, Zwi A, Lozano RJWH. et al, Abuse of the elderly in World Report on violence and health. 2002.

[5] WHO. The Toronto Declaration on the Global Prevention of Elder Abuse. Geneva: World Health Organization, Availablel at; http://apps.who.int/iris/bitstream/10665/42495/1/9241545615_eng.pdf.; 2002.

[6] American College of Healthcare Executives, The National Patient Safety Foundation, Institute for Healthcare Improvement. Leading a culture of safety. A blueprint for success. 2017.

[7] Sokol-Hessner L, Folcarelli PH, Annas CL, Brown SM, Fernandez L, Roche SD (2018). A road map for advancing the practice of respect in health care: the results of an interdisciplinary modified Delphi consensus study. Jt Comm J Qual Patient Saf.

[8] Pilbeam C, Doherty N, Davidson R, Denyer D (2016). Safety leadership practices for organizational safety compliance: developing a research agenda from a review of the literature. Saf Sci.

[9] Nakrem S, Vinsnes AG, Harkless GE, Paulsen B, Seim A (2009). Nursing sensitive quality indicators for nursing home care: international review of literature, policy and practice. Int J Nurs Stud.

[10] National Patient Safety Foundation. Free from Harm, Accelrating Patient Safety Improvment Fifteen Year after To Err is Human (NPSF). Boston: Available at; www.npsf.org: National Patient Safety Foundation (NPSF), ; 2015.

[11] Phelan A (2015). Protecting care home residents from mistreatment and abuse: on the need for policy. Risk Manag Healthc Policy.

[12] Working Group on Elder Abuse. Protecting our future, Report: Dublin, . The Stationery Office. 2002.

[13] Drennan J, Lafferty A, Treacy MP, Fealy G, Phelan A, Lyons I, et al Older people in residential care settings: results of a national survey of staff-resident interactions and conflicts. 2012.

[14] Bužgová R, Ivanová K (2009). Elder abuse and mistreatment in residential settings. Nurs Ethics.

[15] Yon Y, Mikton CR, Gassoumis ZD, Wilber KH (2017). Elder abuse prevalence in community settings: a systematic review and meta-analysis. Lancet Glob Health.

[16] Lachs MS, Teresi JA, Ramirez M, van Haitsma K, Silver S, Eimicke JP (2016). The prevalence of resident-to-resident elder mistreatment in nursing homes. Ann Intern Med.

[17] Rosen T, Pillemer K, Lachs M (2008). Resident-to-resident aggression in long-term care facilities: an understudied problem. NHI Aggression Violent Behav.

[18] Pillemer K, Chen EK, Van Haitsma KS, Teresi J, Ramirez M, Silver S (2012). Resident-to-resident aggression in nursing homes: results from a qualitative event reconstruction study. Gerontologist.

[19] Castle N, Ferguson-Rome JC, Teresi JA (2015). Elder abuse in residential long-term care: an update to the 2003 National Research Council report. J Appl Gerontol.

[20] Lafferty A, Treacy MP, Fealy G, Drennan J, Lyons I (2012). Older people’s experiences of mistreatment and abuse.

[21] World Health Organization (2002). Missing voices; views of Oler persons on elder abuse, Geneva; 2002.

[22] Cilliers P (2002). Complexity and postmodernism: understanding complex systems. London Routledge.

[23] Anderson RA, Issel LM, McDaniel RR (2003). Nursing homes as complex adaptive systems: relationship between management practice and resident outcomes. Nurs Res.

[24] Ostaszkiewicz J. A conceptual model of the risk of elder abuse posed by incontinence and care dependence. Int J Older People Nursing. 2017:1–11. 10.1111/opn.12182 29218819

[25] Helvik A-S, Selbæk G, Benth JŠ, Røen I, Bergh S (2018). The course of neuropsychiatric symptoms in nursing home residents from admission to 30-month follow-u. PLoS One.

[26] Selbaek G, Kirkevold O, Engedal K (2008). The course of psychiatric and behavioral symptoms and the use of psychotropic medication in patients with dementia in Norwegian nursing homes--a 12-month follow-up study. Am J Geriatr Psychiatry.

[27] Malmedal W, Hammervold R, Saveman B-I (2014). The dark side of Norwegian nursing homes: factors influencing inadequate care. J Adult Prot.

[28] Lachs MS, Rosen T, Teresi JA, Eimicke JP, Ramirez M, Silver S (2013). Verbal and physical aggression directed at nursing home staff by residents. J Gen Intern Med.

[29] Goergen T (2004). A multi-method study on elder abuse and neglect in nursing homes. J Adult Prot.

[30] Nerenberg L (2007). Elder abuse prevention: emerging trends and promising strategies.

[31] Brady GP, Cummings GG (2010). The influence of nursing leadership on nurse performance: a systematic literature review. J Nurs Manag.

[32] Braithwaite J, Herkes J, Ludlow K, Testa L, GJBO L (2017). Association between organisational and workplace cultures, and patient outcomes: systematic review. BMJ Open.

[33] Pickering CEZ, Nurenberg K, Schiamberg L (2017). Recognizing and responding to the "toxic" work environment: worker safety, patient safety, and abuse/neglect in nursing homes. Qual Health Res.

[34] HOD. (Helse- og omsorgsdepartementet) [Ministry of Health and Care Service] (2013) Kvalitet og pasientsikkerhet 2013 [Quality and patient safety 2013] (St.meld. nr. 11 2014–2015). Oslo: Departementenes servicesenter.2013.

[35] Northouse PG. (2018). Leadership: theory and practice. Los Angeles; Sage publications.

[36] Health and Safety Commission (1993). Third report: organizing for safety.

[37] World Health Organization (2008). A global response to elder abuse and Negelct: building primary health care capacity to Deal with the problem worldwide.

[38] Cooper C, Selwood A, Livingston G (2009). Knowledge, detection, and reporting of abuse by health and social care professionals: a systematic review. Am J Geriatr Psychiatry.

[39] Radermacher H, Toh YL, Western D, Coles J, Goeman D, Lowthian J (2018). Staff conceptualisations of elder abuse in residential aged care: a rapid review. Aust J Ageing.

[40] Boeije H (2002). A purposeful approach to the constant comparative method in the analysis of qualitative interviews. J Qual Quantity.

[41] Charmaz K (2006). Constructing grounded theory: a practical guide through qualitative analysis. London; sage publications.

[42] Krueger RA, Casey MA (2014). Focus groups: a practical guide for applied research.

[43] SSB. Statistisk Sentralbyrå. [Statistics Norway](2019). Nøkkeltall for helse i Norge [Key number related to health care in Norway]. Available from: https://www.ssb.no/helse/nokkeltallhttps://www.ssb.no/helse/nokkeltall: Statistisk Sentralbyrå SSB.

[44] HOD. Helse-og omsorgsdepartementet [Ministry of health and care services]. (2014). Egenbetaling for kommunale tjenester i og utenfor instiusjon [User fees for primary care in and outside institution]. [updated 18.12.2014]: https://www.regjeringen.no/no/tema/helse-og-omsorg/helse%2D%2Dog-omsorgstjenester-i-kommunene/innsikt/egenbetaling-i-og-utenfor-institusjon/id434597/.

[45] HOD. Helse-og omsorgsdepartementet [Ministry of health and care services]. Forskrift om ledelse og kvalitetsforbedring i helse og omsorgstjenesten [Regulation of management and quality improvement in health care services] no. 1036; Amendment 01.01.2017. 2017 https://lovdata.no/dokument/LTI/forskrift/2016-10-28-1250.

[46] HOD. Helse- og omsorgsdepartementet [Ministry of health and care services]. Forskrift for sykehjem og boform for heldøgns omsorg og pleie [Regulation for nursing homes and residential care] (Updated 01. july 2013) no. 708; Amendment 01.01.1989. 1989. https://lovdata.no/dokument/SF/forskrift/1988-11-14-932.

[47] Trompetter H, Scholte R, Westerhof G (2011). Resident-to-resident relational aggression and subjective well-being n assisted living facilities. Ageing Mental Health.

[48] Matsuda O (2007). An assessment of the attitudes of potential caregivers toward the abuse of elderly persons with and without dementia. Int Psychogeriatrics.

[49] Kales HC (2014). Management of neuropsychiatric symptoms of dementia in clinical settings: recommendations from a multidisciplinary expert panel. JAGS.

[50] Lichtwarck B, Selbaek G, Kirkevold O, Rokstad AMM, Benth JS, Lindstrom JC (2018). Targeted interdisciplinary model for evaluation and treatment of neuropsychiatric symptoms: a cluster randomized controlled trial. Am J Geriatr Psychiatry.

[51] Lichtwarck B, Myhre J, Goyal AR, Rokstad AMM, Selbaek G, Kirkevold Ø, et al. Experiences of nursing home staff using the targeted interdisciplinary model for evaluation and treatment of neuropsychiatric symptoms (TIME)–a qualitative study. Aging Ment Health. 2018:1–10. 10.1080/13607863.2018.1464116 29669442

[52] Utley-Smith Q, Colón-Emeric CS, Lekan-Rutledge D, Ammarell N, Bailey D, Corazzini K (2009). The nature of staff-family interactions in nursing homes: staff perceptions. J Aging Stud.

[53] Vinsnes AG, Nakrem S, Harkless GE, Seim A (2012). Quality of care in Norwegian nursing homes–typology of family perceptions. J Clin Nurs.

[54] Cooper C, Dow B, Hay S, Livingston D, Livingston G (2013). Care workers' abusive behavior to residents in care homes: a qualitative study of types of abuse, barriers, and facilitators to good care and development of an instrument for reporting of abuse anonymously. Int Psychogeriatr.

[55] Jones A, Kelly D (2014). Whistle-blowing and workplace culture in older peoples' care: qualitative insights from the healthcare and social care workforce. Sociol Health Illn.

[56] Ree E, Wiig S (2019). Employees’ perceptions of patient safety culture in Norwegian nursing homes and home care services. BMC Health Serv Res.

[57] Siegel EO, Young HM, Zysberg L, Santillan V (2015). Securing and managing nursing home resources: director of nursing tactics. Gerontologist.

