# Supplementary material for: Elder abuse and neglect: an overlooked patient safety issue. A focus group study of nursing home leaders’ perceptions of elder abuse and neglect
Source: BMC Health Serv Res. 2020 Mar 12;20:199. doi: 10.1186/s12913-020-5047-4 (PMC7069163; doi:10.1186/s12913-020-5047-4)
Supplement: Supplementary file 2 — Additional file 2. Interview guide. [file 12913_2020_5047_MOESM2_ESM.docx]

**Additional file 2 Interview guide**

| **Topic** | **Key questions** |
| --- | --- |
| Introduction | Can you describe what you will define as abuse and neglect in nursing homes? |
| Your experiences of elder abuse and neglect | Within these situations (fig 1), and these categories; *physical abuse, psychological abuse, financial abuse, sexual abuse and neglect,* can you describe your experience of elder abuse and neglect? |
| Communication of elder abuse and neglect | Can you describe how you get knowledge about situations of elder abuse and neglect in the nursing home?  What do you think are barriers and enablers to report elder abuse and neglect? |
| How to follow up on elder abuse and neglect | When you get knowledge about situations of elder abuse and neglect, how do you follow it up?  What do you do to prevent it from happening again? |
| Closure | Do you have anything to add that has not been mentioned?  How did you experience participating in this focus group? |

Note: The result from the topic 2 and 3 in the interview guide: ‘Communication of situations’ and ‘How to follow up on abuse’, will be reported subsequently in a data triangulation comparative study, that combines the focus groups interviews of care managers with individual interviews of nursing home directors.
